# Network Dynamics and Evolutionary Drivers of HIV Drug Resistance in Eastern China, from 2022 to 2024

**DOI:** 10.3390/v17111516

**Published:** 2025-11-19

**Authors:** Dongqing Cao, Hui Xing, Yi Feng, Jiafeng Zhang, Liangkang Zhou, Zhuojing Jiang, Jinkun Chen, Tingting He

**Affiliations:** 1 Shaoxing Center for Disease Control and Prevention, Shaoxing 312000, China; sugus33439980@163.com (D.C.);; 2 State Key Laboratory of Infectious Disease Prevention and Control, National Center for AIDS/STD Control and Prevention (NCAIDS), Chinese Center for Disease Control and Prevention (China CDC), Beijing 102206, China; 3 Department of HIV/AIDS and STDs Control and Prevention, Zhejiang Provincial Center for Disease Control and Prevention, Hangzhou 310051, China

**Keywords:** HIV/AIDS, pretreatment HIV-drug resistance, acquired drug resistance, drug mutation, transmission networks, molecular epidemiology

## Abstract

The increasing prevalence of HIV drug resistance poses a significant challenge. This study aimed to investigate the epidemiological dynamics and molecular characteristics of pretreatment drug resistance (PDR) and acquired drug resistance in Shaoxing, Eastern China. Methods: From 2022 to 2024, 571 newly diagnosed HIV-infected individuals and 119 individuals with antiretroviral treatment failure were enrolled. Molecular transmission networks and Bayesian analysis were employed to identify key drug-resistant clusters and trace their origins. Results: The overall PDR prevalence was 14.4% (85/571). PDR to non-nucleoside reverse transcriptase inhibitors (NNRTIs) was 9.8% (56/571), significantly higher than to NRTIs (1.1%, 6/571) and PIs (3.7%, 21/571) (*χ*^2^ = 50.014, *p* < 0.001). Molecular network analysis identified large clusters harboring K103N and Q58E resistance mutations within the CRF07_BC subtype. Bayesian analysis estimated their introduction into Shaoxing from Guangdong Province around 2016 and 2017, respectively. Integrated network analysis revealed close linkages between virological failure and newly diagnosed cases, highlighting the role of treatment failure in resistance transmission. Conclusion: Targeted interventions against specific subtypes and transmission clusters, alongside continuous resistance surveillance, are essential to curb the spread of drug-resistant HIV and optimize ART regimens.

## 1. Introduction

The high genetic variability of HIV, together with the widespread implementation of antiretroviral therapy (ART), has contributed to an increasing prevalence of drug resistance. This increase is attributable to high-frequency viral mutations and issues with treatment nonadherence. Furthermore, the spread of drug-resistant strains not only compromises treatment success but also increases the risk of transmitting resistant viruses. As the epidemic evolves, both HIV pretreatment drug resistance (PDR) and acquired drug resistance (ADR) have emerged as major obstacles to effective disease management. In this context, the surveillance of PDR is imperative for informing the selection of initial ART regimens and for understanding the transmission dynamics of resistant HIV-1 among treatment-naïve individuals. Likewise, monitoring ADR is paramount for adjusting treatment strategies and preventing the accumulation of resistance.

Globally, the prevalence of PDR to nonnucleoside reverse transcriptase inhibitors (NNRTIs), such as efavirenz, has exceeded the 10% international warning threshold in certain regions [1,2]. A particularly sharp increase has even been observed in some high-risk populations, including neonates infected through mother-to-child transmission and women of reproductive age [3]. Mirroring these international trends, although China’s two-decade-long provision of free HIV treatment has significantly reduced AIDS-related mortality, the challenge of viral drug resistance has become increasingly severe. Multiple studies have reported the increasing detection of resistance mutations associated with NNRTIs, nucleoside reverse transcriptase inhibitors (NRTIs), and protease inhibitors (PIs) among newly diagnosed cases in China [4,5,6].

According to the National Center for AIDS/STD Control and Prevention, China CDC, the number of reported surviving cases in China is increasing, and by December 31, 2024, the population of documented PLWHA in the country had reached 1.355 million [7]. In China, the overall prevalence rate of PDR remains at 7.4% [4]. The incidence rates of PDR and ADR mutations are increasing, yet there are significant differences among different regions [8,9,10,11,12]. As HIV evolves in different regions, continuous monitoring of drug resistance trends has become increasingly important for ensuring the long-term efficacy of antiretroviral therapy and preventing the further spread of drug-resistant strains.

A high rate of PDR of 14.6% has been previously reported in Shaoxing, a developed city in eastern China [13], highlighting the critical need to investigate the transmission dynamics of drug-resistant HIV strains. In this study, molecular transmission network monitoring was integrated with Bayesian phylogenetic analysis to trace the dissemination pathways of drug-resistant HIV. By incorporating geographic and demographic data to increase network resolution, we identified key transmission clusters and core populations. Furthermore, time-calibrated phylogenetic trees were constructed to infer the evolutionary timeline of the virus, including the time to the most recent common ancestor (tMRCA) and evolutionary rates. Through the synthesis of multisource data, we systematically examined the drivers underlying the emergence of drug resistance and determined the origins of transmitted drug-resistant strains in Shaoxing. These findings provide a crucial scientific basis for developing targeted, localized intervention strategies.

## 2. Materials and Methods

### 2.1. Study Population and Data Collection

From 2022 to 2024, 640 (85.3%) samples were obtained from 750 newly confirmed HIV- and treatment-naive patients, and 142 samples were obtained from individuals with ART failure by the Shaoxing Center for Disease Control and Prevention (Shaoxing CDC). The plasma was separated on the day after sampling and stored at −80 °C. The inclusion criteria for new individuals were as follows: (1) aged 16 years and older; (2) newly diagnosed with HIV-1; and (3) had not received ART before enrolment. The criteria for ART failure were as follows: (1) had been treated for 6 months and (2) had an HIV-1 viral load greater than 1000 copies/mL. Sociodemographic information was obtained from the National Center for AIDS/STD Control and Prevention (NCAIDS) information systems.

### 2.2. Laboratory Testing and Sequencing

Viral RNA was extracted from samples using an NP968-C automated nucleic acid extraction system(Tianlong, Xian, China) in conjunction with a Tianlong RNA Extraction Kit. This was followed by amplification of a partial pol gene region (HXB2: 2253–3306) [14] through a one-step RT–PCR and nested PCR approach with a PrimeScript kit (Takara, Dalian, China). Purification and Sanger sequencing of the positive PCR products were conducted by TsingKe Bio-Tech Co. Prior to analysis, all sequences were assembled and edited in Sequencher v5.4.6 software (Genecodes, Ann Arbor, MI, USA), and those meeting exclusion criteria (≥5% ambiguous bases or <1000 bp length) were discarded.

### 2.3. HIV Subtype Analysis

The assembled sequences were aligned and analyzed using BioEdit 7.2 software alongside reference sequences retrieved from the Los Alamos National Laboratory (LANL) HIV sequence database (https://www.hiv.lanl.gov) (accessed on 1 August 2025). For subtype classification, maximum-likelihood phylogenetic trees were built with IQ-TREE 2.0.6, implementing the GTR + G + I nucleotide substitution model. A sequence was assigned to a subtype if it clustered with reference sequences with a bootstrap support value of ≥70%. Those suspected of being recombinants based on initial phylogenetic analysis were further investigated for intersubtype recombination using SimPlot v3.5.1.

### 2.4. Drug Resistance Analysis

HIV-1 pol gene sequences were submitted to the Stanford HIVdb program (https://hivdb.stanford.edu/) (accessed on 1 August 2025) for genotypic resistance analysis. Drug resistance mutations (DRMs) and their associated resistance levels were interpreted according to the Stanford HIV Drug Resistance Database algorithm. Viruses exhibiting low-level resistance or higher were classified as resistant.

### 2.5. Genetic Network Analysis

Genetic distances were computed under the tamura-nei 93 model using HIV-TRACE After evaluating thresholds from 0.0025 to 0.015 (increment 0.0025), we selected GD = 0.0125, which yielded the maximum number of transmission clusters, for all subsequent analyses. The inferred molecular transmission network was visualized in Cytoscape v3.9.0. in this study, individuals with a mean link count exceeding 4.7 were classified as high-risk transmitters.

### 2.6. Bayesian Phylogenetic Analysis

The reference sequences of CRF07_BC from other regions in China were downloaded from the HIV sequence database (https://www.hiv.lanl.gov) (accessed on 10 August 2025) on the basis of their high homology (>98.0%) with the sequences in drug-resistant transmission clusters. Repetitive sequences and sequences without time and regional source identifiers were excluded. The maximum likelihood (ML) phylogenetic tree was constructed through IQ Tree 2.0.6 in the GTR + G + I nucleotide substitution model. BEAST v.1.8.4 under an uncorrelated relaxed clock model and a Bayesian Skygrid demographic model [15] were used to perform Bayesian evolutionary analysis. The BEAST analysis was performed using Markov Chain Monte Carlo (MCMC) runs of 100 million generations. Every 1000 iterations were sampled, and the first 10% were discarded as burn-in. Convergence, defined as an effective sample size (ESS) ≥ 200, was determined in Tracer v.1.6. Maximum clade credibility (MCC) trees were generated using TreeAnnotator v1.8.4 and visually edited in FigTree v1.4.4.

### 2.7. Statistical Analysis

Categorical variables were used to represent all collected data, which are presented as frequencies and percentages. Intergroup comparisons were performed using Chi-square or Fisher’s exact tests. To identify factors associated with transmission clustering, we conducted both univariate and multivariate logistic regression analyses. Variables showing significance at *p* < 0.10 in univariate analysis were included in the multivariable model. All statistical analyses were performed using IBM SPSS Statistics 26.0 (IBM Corp., New York, NY, USA), with two-sided *p* values < 0.05 considered statistically significant.

## 3. Results

### 3.1. Patient Demographic Characteristics

A total of 713 qualified sequences were obtained in this study, including 571 newly diagnosed HIV cases and 142 HIV ART-failure cases. Of these, 23 sequences were deleted because of duplication. Therefore, the numbers of participants with newly confirmed HIV infection and ART failure were n1 = 571 and n2 = 119, respectively. As shown in Table 1, among those with newly confirmed HIV infections, the majority were male (83.0%, 474/571), younger individuals (<50 y, 58.0%, 331/571), of Han ethnicity (92.8%, 530/571), married (52.5%, 300/571), had a junior middle school education (37.3%, 213/571), and had acquired HIV through heterosexual contact (67.8%, 387/571). A total of 12 subtypes were detected. CRF07_BC was the predominant subtype, accounting for 44.8% (256/571) of the sequences, followed by CRF01_AE (30.5%, 174/571), CRF08_BC (14.5%, 83/571), CRF85_BC (3.2%, 18/571), CRF55_01B (2.6%, 15/571), B (1.6%, 10/571), C (0.4%, 2/571), CRF87_cpx (0.4%, 2/571), CRF57_BC (0.2%, 1/571), URF (CRF01_AE/CRF07_BC) (1.4%, 8/571), URF (CRF01_AE/C) (0.2%, 1/571), and URF (B/C) (0.2%, 1/571). The proportions of individuals who experienced ART failure were similar in terms of sex, age, ethnicity, educational level, marital status and infection route (Table 1).

### 3.2. Drug Resistance Mutation Analysis

**Figure 1:**
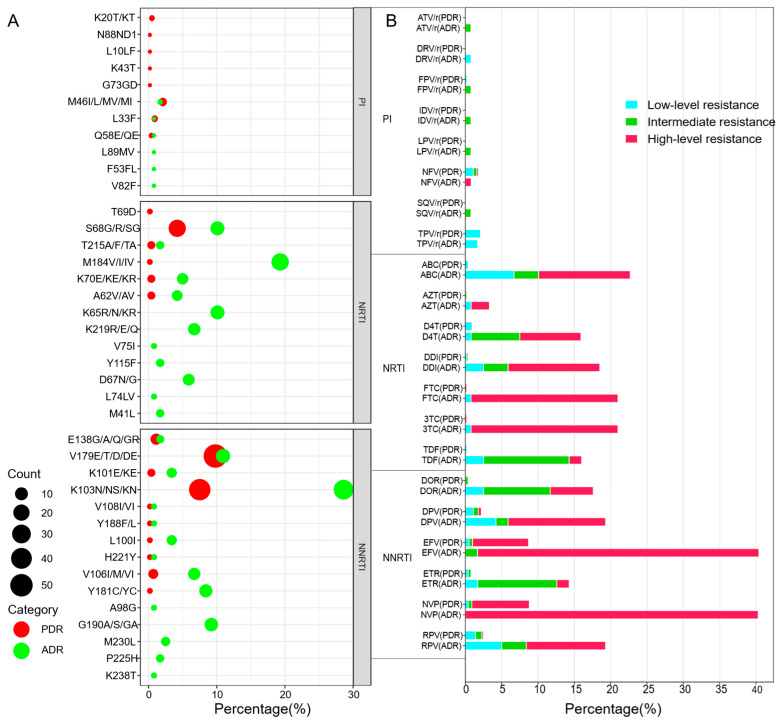
(**A**): The frequency of drug-resistant mutations to PIs, NRTIs and NNRTIs in 571 newly reported HIV-infected individuals and 119 HIV ART failure individuals. (**B**): Level of HIV-1 PDR associated mutations to different ART drug among 571 newly diagnosed HIV/AIDS patients and ADR to HIV treatment failure cases. PI, Protease inhibitor; NRTI, Nucleoside reverse transcriptase inhibitor; NNRTI, Non-nucleoside reverse transcriptase inhibitor; ATV/r: Atazanavir/ritonavir; FPV/R: Fosamprenavir/ritonavir; IDV/r: Indinavir/ritonavir; LPV/r: Lopinavir/ritonavir; NFV: Nelfinavir; SQV/r: Saquinavir/ritonavir; TPV/r: Ritonavir/ritonavir; ABC: Abacavir; AZT: Zidovudine; D4T: Stavudine; DDI: Didanosine; FTC: Emtricitabine; 3TC, Lamivudine; TDF, Tenofovir; DOR, Doravirine; EFV, Efavirenz; ETR: Etravirine; NVP: Nevirapine; RPV: Rilpivirine.

The prevalence of PDR among newly diagnosed patients was 14.4% (82/571). Eight PI resistance-associated mutation patterns, 6 NRTI mutation patterns, and 10 NNRTI mutation patterns were identified. The overall prevalence of HIV-1 PDR to NNRTIs (9.8%, 56/571) was much greater than that to NRTIs (1.1%, 6/571) and PIs (3.7%, 21/571) (*χ*^2^ = 50.014, *p* < 0.001). Among the PI agents, resistance to tipranavir (TPV/r) was the most common (2.1%, 12/571), which was related mainly to the Q58E/QE mutation (2.1%, 12/571) (Figure 1). For NNRTI agents, the most frequent mutation was K103N/KN/NS (7.5%, 43/571), which was responsible for the greatest proportion of high-level resistance to nevirapine (NVP) and efavirenz (EFV) (57.5%).

The overall prevalence of ADR among individuals with ART failure was 45.4% (54/119). The frequencies of drug resistance-associated mutations to NNRTIs, NRTIs, and PIs were 42.0% (50/119), 23.5% (28/119), and 2.5% (3/119), respectively, demonstrating statistically significant differences (*χ*^2^ = 52.985, *p* < 0.001). The predominant drug resistance mutations among the NRTI-resistant strains were M184I/V (19.3%, 23/119) and S68G/R/SG (10.1%, 12/119), whereas among the NNRTI-resistant strains, K103N/NS/KN (28.6%, 34/119) and V179E/T/D/DE (10.9%, 13/119) were the most frequent.

Of the 54 patients who experienced treatment failure and demonstrated resistance, 41 had been on a regimen of 2NRTIs + NNRTI. The most common regimen was 3TC + EFV + TDF, accounting for 70.7% (29/41) of these cases; other combinations included AZT + 3TC + EFV, 3TC + AZT + NVP, 3TC + TDF + NVP, and ABC + 3TC + TDF. Eight patients (14.8%, 8/54) had received 2NRTIs + INSTI, such as 3TC + TDF + LPV/r, AZT + 3TC + LPV/r, or BIC + FTC + TAF. Two patients (3.7%, 2/54) were on a NRTI + INSTI (3TC + DTG) therapy. Overall, 44 (81.5%) of the 54 patients carried resistance mutations directly corresponding to their prescribed drug regimens (Table 2).

No HIV-1 strains with PDR mutations to three classes of drugs were found in this study. Only 1 (2.9%, 1/82) strain harbored two classes of drug resistance mutations in PDR, while 27 (50%, 27/54) strains were resistant to two classes of drugs, and 27 (50%, 27/54) strains were resistant to three classes in ADR.

### 3.3. HIV Molecular Transmission Network Analysis

**Figure 2:**
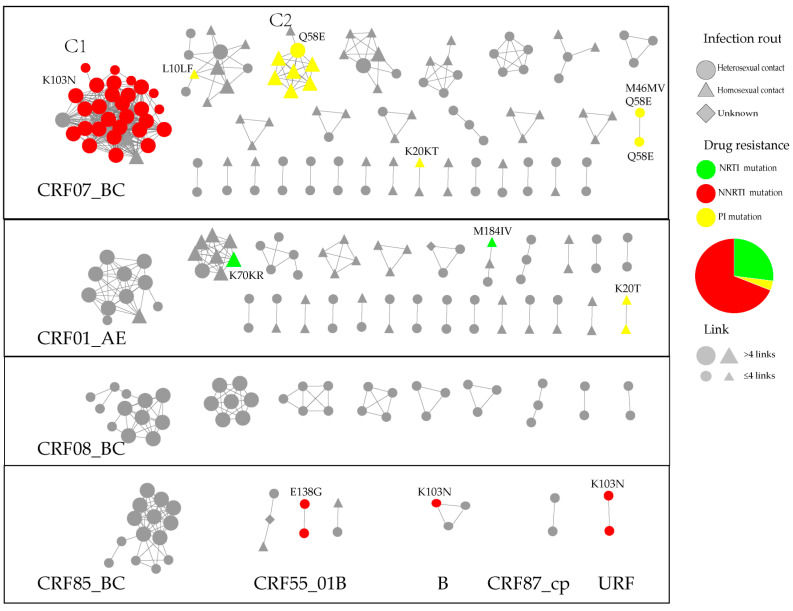
The molecular network of the newly reported HIV infections in Shaoxing. The clusters were partitioned according to the subtypes. Gray nodes represent the individuals with no PDR. Colored nodes represent different PDR mutations. The shape square, circle, triangle and diamond represent the route of HIV infection through heterosexual contact, homosexual contact, and unknown, respectively.

At the optimal GD of 0.0125 substitutions/site, 269 individuals (47.1%, 269/571) formed 71 clusters (mean 3.8, median 2.0, range 2–32, interquartile range 2–3) and 632 edges (mean 4.7, median 2.0, range 1–29, interquartile range 1–6) (Figure 2). Among the 71 clusters, the subtypes CRF07_BC formed 30 clusters (46.8%, 126/269), and CRF01_AE (27.5%, 74/269), CRF08_BC (15.2%, 41/269), CRF85_BC (5.2%, 14/269), CRF55_01B (2.6%, 7/269), B (1.1%, 3/269), CRF87_cpx (0.7%, 2/269) and URF (CRF01_AE/CRF07_BC) (0.7%, 2/269) formed clusters of 25, 9, 1, 3, 1, 1, and 1, respectively. In the molecular network, clusters with more than 3.8 nodes on average were defined as large clusters.

There were 48 individuals with PDR in the network. The primary subtype distributed was CRF07_BC (81.3%, 39/48), while others included CRF01_AE (8.3%, 4/48), CRF55_01B (4.2%, 2/48), B (2.1%, 1/48), and URF (CRF01_AE/CRF07_BC) (4.2%, 2/48). K103N (64.6%, 31/48) was the most common NNRTI (68.8%, 33/48) mutation, while others included E138G (4.2%, 2/48). Resistance mutations for PIs (27.1%, 13/48) included Q58E (18.8%, 9/48), K20T (6.3%, 3/48), and L10LF (2.1%, 1/48), while NRTIs (4.2%, 2/48) included M184IV (2.1%, 1/48) and K70KR (2.1%, 1/48). K103N and Q58E, with the highest occurrence rates, were present mainly in two large clusters, and they also appeared in other subtypes or other clusters. The largest cluster with K103N was mainly the heterosexual transmission (93.8%, 30/32) cluster of over 50 years old (93.8%, 30/32), and the large Q58E cluster was mainly the MSM (87.5%, 7/8) cluster of under 25 years old (87.5%, 7/8). There were 1–2 cases of other PDR mutations in each class, and K20T/KT, M184IV, and E138G were in small clusters; by contrast, L10LF and K70KR were located in the large clusters of CRF07_BC and CRF01_AE, respectively.

Multivariate logistic regression analysis revealed that patients aged < 50 years had significantly lower odds of clustering than elderly patients did (AOR = 0.551, 95% CI 0.374–0.811). No statistically significant differences were observed for other variables, such as sex, ethnicity, marital status, HIV-1 subtype, and education (all *p* > 0.05)(Table 3).

**Figure 3:**
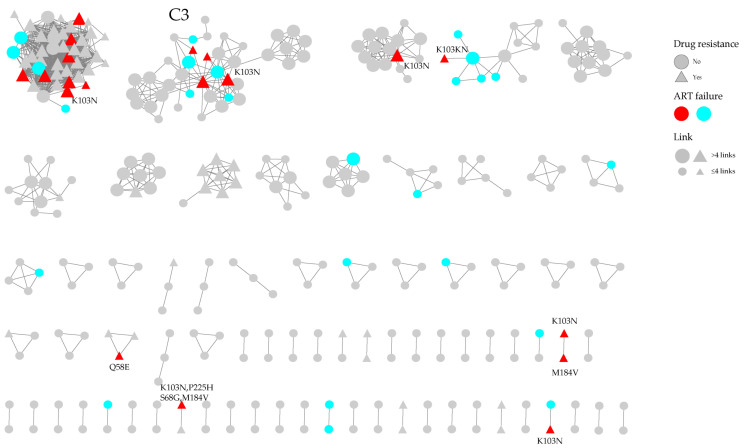
The molecular network combining the HIV infections with ART failure and newly confirmed HIV infections in Shaoxing. Gray nodes represent the newly reported HIV infections, colored nodes represent the ART failure cases, respectively. Colored nodes represent different PDR mutations. The triangle and circle represent the individuals with DR and with no DR, respectively.

After the sequences of 119 ART-failure cases were incorporated, a total of 44 cases within the molecular network were associated with newly diagnosed cases in 17 clusters (Figure 3). The 44 patients were treated for 2 to 16 years, with a mean duration of 7.2 years. Among them, 19 were drug-resistant viral strains, while the rest were nondrug resistant. In addition, 95.5% (42/44) of the patients had a viral load exceeding 10,000 copies/mL, whereas 20.5% had a viral load below 10,000 copies/mL. Three large clusters were associated with more than three cases of ART failure. The largest cluster, C1, increased by 12 cases, 8 with K103N. Additionally, 11 newly identified molecular clusters were linked to a treatment failure case, three of which were drug-resistant strains. Nine treatment failure CRF08_BC cases were associated with five newly diagnosed clusters, forming the large Cluster C3, which comprises 39 nodes.

### 3.4. Bayesian Evolutionary Analysis of the Drug-Resistant Transmission Clusters

**Figure 4:**
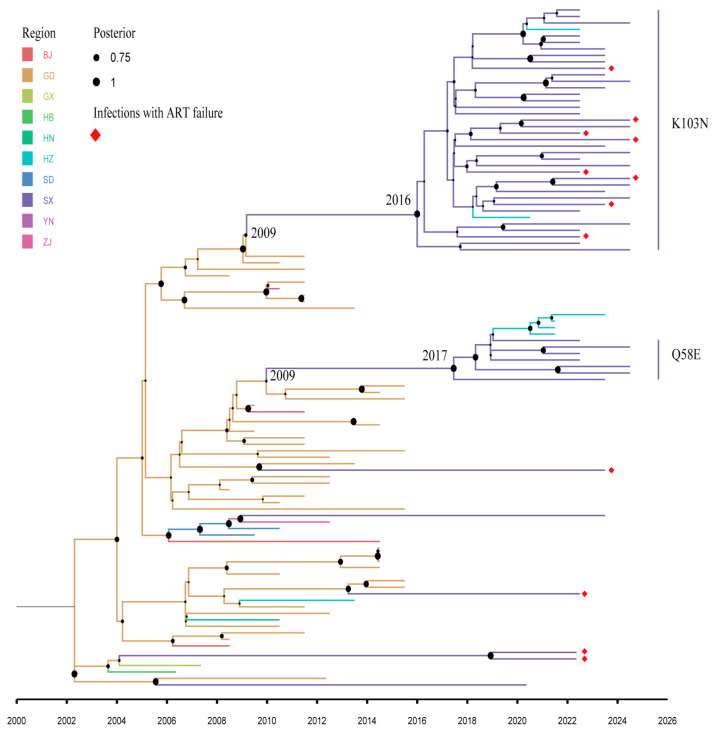
Maximum evolutionary confidence (MCC) tree of patients with CRF07_BC resistant subtype in Shaoxing city from 2022–2024. Bayesian analyses were performed using BEAST v1.8.4. The branch lengths reflect the evolutionary time, and labeled with posterior probability. The corresponding time scale was marked at the bottom of the MCC tree; different colors of branches indicate that the formed clades contain reference sequences from different provinces/cities in China. Red diamond represents ART failure individuals.

Bayesian evolutionary analysis was performed on 105 sequences, including those of CRF07_BC drug-resistant strains within the molecular network and their homologous sequences. The most frequent mutations in CRF07_BC within the network were K103N and Q58E, which probably originated from Guangdong Province, China (Figure 4). They emerged in 2009–2010. Since 2017, two drug-resistant strains have spread rapidly in the local area and further disseminated to the surrounding Hangzhou region. The average evolution rate of the clusters was 1.025 × 10^−3^ nucleotide substitutions/site/year (95% HPD: 7.75 × 10^−4^–1.30 × 10^−3^).

## 4. Discussion

Our study revealed that the prevalence of PDR to HIV-1 in the Shaoxing region was significantly higher than the national average in China [4], whereas the ADR was lower [16]. Resistance mutations associated with NNRTIs were markedly more prevalent than those linked to NRTIs and PIs were, suggesting that NNRTI resistance serves as the principal driver of local drug-resistant HIV transmission. In PDR patients, the most commonly detected NNRTI-associated mutations were V179E/T/D/DE (9.8%) and K103N/NS/KN (7.5%), whereas S68G/R/SG (4.2%) was the most frequent NRTI mutation. Among ADR cases, NNRTI resistance mutations were similarly dominated by K103N/NS/KN (28.6%) and V179E/T/D/DE (10.9%), whereas the predominant NRTI mutations included M184V/I/IV (19.3%), K65R/N/KR (10.1%), and S68G/R/SG (10.1%). This mutation profile aligns with the national resistance trend, and the high frequency of these mutations reflects the impact of current first-line antiretroviral regimens in China. Of particular significance is the low genetic barrier of NNRTIs, wherein a single mutation can confer high-level cross-resistance to multiple drugs within this class. From a global perspective, the high prevalence of NNRTI resistance remains a widespread public health challenge. According to the WHO report “HIV Drug Resistance Report 2021,” PDR to NNRTIs in the Americas and Africa reached 16.7% and 15.4%, respectively, between 2014 and 2020, with no substantial improvement observed in the following years [17]. These findings underscore the urgency and justify the ongoing global transition from EFV/NVP based regimens to those centered on dolutegravir (DTG). Although the NNRTI resistance rate in Shaoxing is lower than that in these high-burden regions, the local resistance profile mirrors broader global trends, highlighting the need for sustained vigilance and tailored interventions within local HIV treatment programs. Notably, a relatively high prevalence of the Q58E/QE mutation (2.1%) was identified among PI-associated resistance mutations. Although this mutation is generally detected at low frequencies nationwide, recent studies have reported an increasing trend in eastern China [18,19,20]. As an accessory resistance mutation for PIs, Q58E confers low-level resistance to tipranavir (TPV). Its elevated frequency in eastern China indicates potential regional transmission clusters, underscoring the need for enhanced surveillance in subsequent monitoring efforts.

CRF07_BC is currently the most prevalent HIV-1 subtype circulating in China [21]. Its widespread transmission across all routes [22] is likely facilitated by its enhanced replicative capacity within the human host [23,24]. In our study, CRF07_BC was the predominant subtype (46.8%, 126/269) within the constructed molecular transmission network. Among these CRF07_BC cases, heterosexual transmission accounted for 61.1% (77/126), whereas homosexual transmission represented 38.9% (49/126). Phylogenetic analysis revealed two major transmission clusters (C1 and C2; Figure 2), primarily associated with specific resistance mutations: K103N for C1 and Q58E for C2. Cluster C1 consisted predominantly of individuals over 50 years old (93.8%, 30/32) reporting heterosexual contact (93.8%, 30/32). In stark contrast, Cluster C2 was characterized by individuals under 25 years old (87.5%, 7/8) and predominantly involved homosexual transmission (87.5%, 7/8). The high genetic similarity within these clusters suggests potential outbreak events. Furthermore, the formation of such distinct clusters indicates that these drug-resistant strains may have acquired transmission fitness, overcoming the potential fitness cost often associated with resistance mutations and enabling efficient human-to-human spread. Viral adaptation may have been achieved through compensatory evolution. Against the backdrop of the widespread use of low genetic barrier drugs, these strains appear to be efficiently transmitted, potentially via individuals experiencing treatment failure who act as transmission sources. These findings highlight the urgent need for targeted interventions to curb the spread of these specific resistant variants.

Within the molecular network comprising all the cases (Figure 3), nine treatment-failure cases of the CRF08_BC subtype linked five smaller clusters of newly diagnosed cases (Figure 2), forming a large molecular cluster designated C3 (Figure 3). Additionally, 11 other newly diagnosed cases were directly connected to treatment-failure cases within the network. Although direct evidence is not available, this highly interconnected topological structure strongly suggests that treatment-failure cases act as a core driver—functioning as “bridges” that integrate disparate transmission chains—and facilitate the clustered spread of drug-resistant viruses.

Moreover, on the basis of the Bayesian evolutionary analysis results (Figure 4), the earliest cases carrying the K103N mutation within molecular Cluster C1 can be traced back to 2016, potentially originating from an introduction from Guangdong Province. This was followed by rapid local expansion between 2017 and 2018, with the number of new infections plateauing after 2021. Within this cluster, eight treatment-failure cases were identified, all of which carried the K103N mutation and were closely interwoven with newly diagnosed cases in the molecular network. Furthermore, subsequent transmission involving viral sequences from Hangzhou was observed.

Guangdong Province, a major economic and demographic hub in China, has been identified by multiple studies as a key epicenter for the nationwide dissemination of various HIV strains. For instance, Gan et al. [15] clearly demonstrated how the CRF55_01B strain spread from Guangdong to other regions along major transportation routes. Our findings further support this pattern, suggesting that the virus may have been introduced to Shaoxing from Guangdong through frequent population mobility, such as labor migration and commercial activities. Treatment-failure patients likely acted as persistent sources of infection, which played a significant role in the ongoing transmission and maintenance of this resistant strain.

Therefore, to effectively prevent the spread of resistant strains, establishing a regional collaborative surveillance network for sharing viral genetic sequence data is crucial. As advocated by Xu et al. [11] in southwestern China, such a network is essential for timely warning and tracking of emerging resistant variants, thereby enabling targeted, region-specific prevention and control measures.

This study inevitably has several limitations. First, the sequence analysis was restricted to partial regions of the POL gene and utilized first-generation sequencing technology, which has a detection threshold of 20% variant frequency. These constraints may have resulted in the omission of low-frequency drug resistance mutations, potentially leading to an underestimation of the overall drug resistance rate. Second, the development of ADR is influenced by multiple factors, with patient treatment adherence being particularly important. However, owing to limitations related to patient privacy, the study duration, and funding, a comprehensive analysis of these factors was not feasible, which may affect the completeness of our interpretation of the drivers of drug-resistant transmission.

## 5. Conclusions

In summary, this study included a systematic analysis of PDR and ADR among newly diagnosed individuals and ART-failure patients in Shaoxing. By integrating molecular transmission network monitoring technology, we elucidated the patterns and relationships of viral transmission within the population. The close relationships between treatment-failure cases and newly diagnosed individuals within the molecular network confirm the significant role of failure cases in propagating drug resistance. CRF07_BC was the primary subtype that formed major transmission clusters. Strains carrying key resistance mutations, such as K103N and Q58E, have established large clusters within specific demographic groups, indicating that resistant viruses have developed efficient local transmission chains with the potential for ongoing spread to surrounding areas. The increasing prevalence of PDR increases the risk of first-line regimen failure from the outset of therapy for a growing number of patients. Consequently, the increase in treatment failures can fuel the further transmission of resistant strains, creating a vicious cycle of “treatment failure → spread of resistant strains → further treatment failures”. Of particular concern is the concentrated transmission of resistant strains among older populations, who may require more tailored treatment regimens because of underlying health complexities. If this trend escalates, it could pose a significant public health threat in China. These findings underscore the urgent need to implement universal pretreatment drug resistance testing and to optimize standard first-line antiretroviral therapy regimens.

## Figures and Tables

**Table 1:** Demographic characteristics of the newly reported HIV infections and HIV infections with ART failure in Shaoxing.

Categories	Newly Reported HIV Infections Number (%)	HIV Infections with ART Failure Number (%)
Total	571	119
Collection time		
2022	189 (33.1)	28 (23.5)
2023	175 (30.6)	40 (33.6)
2024	207 (36.3)	51 (42.9)
Sex		
Male	474 (83.0)	101 (85.0)
Female	97 (17.0)	18 (15)
Age(years)		
≤25	72 (12.6)	23 (19.3)
26–39	148 (25.9)	29 (24.3)
40–49	111 (19.4)	19 (16.0)
50–59	145 (25.4)	29 (24.4)
≥60	95 (16.6)	19 (16.0)
Ethnicity		
Ethnic Han	530 (92.8)	96 (96.4)
Others	41 (7.2)	4 (3.4)
Married status		
Single	174 (30.5)	30 (25.2)
Married	300 (52.5)	61 (51.3)
Divorced/Widowed	94 (16.5)	28 (23.5)
Unknow	3 (0.5)	0
Education		
Junior college or above	76 (13.3)	18 (15.1)
High or technical secondary school	120 (21.0)	22 (18.5)
Junior middle school	213 (37.3)	43 (36.1)
Primary school/illiterate	162 (28.4)	36 (30.3)
Subtype		
CRF07_BC	256 (44.8)	45 (37.8)
CRF01_AE	174 (30.5)	40 (33.6)
CRF08_BC	83 (14.5)	19 (16.0)
CRF85_BC	18 (3.2)	4 (3.4)
Others	40 (7.0) ^a^	11 (9.2) ^b^
Sexual contact		
Heterosexual	387 (67.8)	85 (71.4)
Homosexual	177 (31.0)	28 (23.5)
IDUs	0	2 (1.7)
Unknow	7 (1.2)	4 (3.4)
With DR		
Yes	82 (14.4)	54 (45.4)
No	489 (85.6)	65 (54.6)
Viral load (Copies/mL)		
1000–10,000	/	36 (30.3)
10,000–50,000	/	38 (31.9)
50,000–100,000	/	15 (12.6)
≥100,000	/	30 (25.2)

^a^: Others were CRF55_01B 15, B 10,C 2,CRF87_cpx 2,CRF57_BC 1, URF(B/C) 1, (CRF01_AE/C) 1, and URF (CRF01_AE/CRF07_BC) 8. ^b^: Others were C 6, CRF55_01B 2, B 3; /: Not detected.

**Table 2:** Resistance patterns associated with ART regimen in 54 patients.

ART Regimen	Cases (N = 54)	Drug Resistance-Related Mutant Genes			Drug Resistance (Level, *n*)		
		PIs	NRTIs	NNRTIs	PIs	NRTIs	NNRTIs
3TC + EFV + TDF	29	Q58E/QE (2)	M184V/I/MV (12), K65R/N/KR (10), S68G/SG (7), K219E/KE (5), A62V (4), D67G/N (2), K70Q/E (2), Y115F (2), L74LV (1), V75I (1)	G190A/S/GA (7), K103N/NS (6), V179D/E (5), Y181C/YC (5), V106M (4), K101E/EQ (2), L100I (2), M230L (2), K221Y (1), K238T (1), P225H (1), V108I (1)	TPV (L, 2)	ABC (H, 9; I, 3: L, 1), AZT (L, 1), D4T (H, 4; I, 7; L, 1), DDI (H, 9; I, 3), FTC (H, 12; L, 1), 3TC (H, 12; L, 1), TDF (H, 1; I, 9; L, 2)	DOR (H, 3;I, 8; L, 2), DPV (H, 10;I, 2; L, 3), EFV (H, 26; I, 1), ETR (H, 2; I, 8; L, 1), NVP (H, 27), RPV (H, 9; I, 2; L, 4)
AZT + 3TC + EFV	6	/	M184V (3), K70R (2), M41L (2), T215F (2), D67G/N (2), K219E/Q (2)	K103N/KN (3), G190S/A (2), V179D/E (2), Y188L (1), P225H (1), Y181C (1), V106I (1), A98G (1)	/	ABC (H, 2; L, 2), AZT (H, 2), D4T (H, 2), DDI (H, 2; L, 1), FTC (H, 3), 3TC (H, 3), TDF (H, 1; I, 1)	DOR (H, 2; I, 1; L, 1), DPV (H, 2; L, 1), EFV (H, 6), ETR (H, 1; L, 1), NVP (H, 6), RPV (H, 2; L, 1)
3TC + AZT + NVP	2	/	D67N (2), M184V (2), K70R (1), T215F (1), K219Q (1)	K103N (2), M230L (1)	/	ABC (H, 1; L, 1), AZT (H, 1), D4T (H, 1), DDI (H, 1; L, 1), FTC (H, 2), 3TC (H, 2), TDF (I, 1)	DOR (H,1), DPV (H,1), EFV (H,2), ETR (I,1), NVP (H,2), RPV (H,1)
3TC + TDF + NVP	2	/	K65R (1), S68G (1), M184V (1), K219E (1)	K103N (2), Y181C (1)	/	ABC (H, 1), D4T (H, 1), DDI (H, 1), FTC (H, 1), 3TC (H, 1), TDF (I, 1)	DPV (H, 1), EFV (H, 2), ETR (I, 1), NVP (H, 2), RPV (H, 1)
ANV + 3TC + TDF	2	/	D67G (1), M184V (1)	K103N/KN (2), E138Q (1)	/	ABC (L, 1), DDI (L, 1), FTC (H, 1), 3TC (H, 1)	DPV (L, 1), EFV (H, 2), NVP (H, 2), RPV (L, 1)
3TC + TDF + LPV/r	3	F53FL (1), M46MI (1), V82F (1)	D67N (1), K70E (1), M184IMV (2)	K103N (2), V179D/E (2)	ATV/r (I, 1),DRV/r (L, 1),FPV/r (I, 1), IDV/r (I, 1), LPV/r (I, 1), NFV (H, 1), SQV/r (I, 1)	ABC (I, 1; L, 1), D4T (I, 1), DDI (I, 1), FTC (H, 2), 3TC (H, 2), TDF (L, 1)	EFV (H, 2), NVP (H, 2)
AZT + 3TC + LPV/r	3	/	S68G (1), M184V (2), K65KR (1)	K103N (3), P225H (1), L100I (1)	/	ABC (H, 1; L, 1), D4T (I, 1), DDI (H, 1), FTC (H, 2), 3TC (H, 2), TDF (I, 1)	DOR (I, 2), DPV (H, 1), EFV (H, 3), ETR (I, 1), NVP (H, 3), RPV (H, 1)
BIC + FTC + TAF	2	/	/	Y181C (1), K103N (1)	/	/	DOR (I, 2), DPV (H, 1), EFV (H, 1; I, 1), ETR (I, 1), NVP (H, 2), RPV (I, 1)
3TC + DTG	3	/	A62V (1), K65R (2), M184V (3), K219E (2), S68SG (1)	K101E (1), V106I (1), Y181C (1), G190S (1), L100I (1), K103N (1)	/	ABC (H, 2), D4T (H, 2), DDI (H, 2), FTC (H, 3), 3TC (H, 3), TDF (I, 2)	DOR (H, 1; I, 1), DPV (H, 2), EFV (H, 2), ETR (H, 1; I, 1), NVP (H, 2), RPV (H, 2)
Unknow	2	/	/	K103N/KN (2)	/	/	EFV ( H, 2), NVP (H, 2)

BIC: Bictegravir; TAF: Tenofovir Alafenamide; DTG: Dolutegravir; H: High-level resistance; I: Intermediate resistance; L: Low-level resistance; /: No drug-resistant mutations or drug resistance was detected.

**Table 3:** Factor associated with clustering of newly diagnosed HIV-1 patients in Shaoxing from 2022 to 2024.

Categories	Newly Diagnosed Cluster Cases (%) (*n* = 269)	Univariate Analysis		Multivariate Analysis	
		OR (95% CI)	*p*	AOR (95% CI)	*p*
Age(years)					
<50	132 (59.1)	1.000		1.000	
≥50	137 (50.9)	0.499 (0.356–0.699)	0.000	0.551 (0.374–0.811)	0.003
Sex					
Male	220 (81.8)	1.000			
Female	49 (18.2)	0.848 (0.548–1.314)	0.461		
Ethnicity					
Ethnic Han	256 (95.2)	1.000		1.000	
Others	13 (4.8)	2.012 (1.020–3.970)	0.044	1.779 (0.894–3.540)	0.101
Married status					
Single	70 (26.0)	1.000		1.000	
Married	156 (58.0)	0.621 (0.426–0.907)	0.014	0.886 (0.573–1.369)	0.586
Divorced/Widowed	41 (15.2)	0.870 (0.524–1.446)	0.591	1.104 (0.646–1.887)	0.717
Unknow	2 (0.7)	0.337 (0.030–3.783)	0.378	0.613 (0.050–7.502)	0.702
Education					
Junior college or above	78 (29.0)	1.000			
High or technical secondary school	109 (40.5)	0.886 (0.589–1.333)	0.562		
Junior middle school	50 (18.6)	1.300 (0.808–2.093)	0.280		
Primary school/illiterate	32 (11.9)	1.277 (0.737–2.213)	0.384		
Subtype					
CRF07_BC	126 (46.8)	1.000			
CRF01_AE	74 (27.5)	1.310 (0.889–1.930)	0.173		
CRF08_BC	41 (15.2)	0.993 (0.605–1.629)	0.977		
Other	28 (10.4)	1.038 (0.587–1.837)	0.897		
Sexual contact					
Heterosexual	184 (68.4)	1.000			
Homosexual	83 (30.9)	1.027 (0.719–1.466)	0.885		
Unknow	2 (0.7)	2.266 (0.434–11.822)	0.332		
PDR					
No	221 (82.2)	1.000		1.000	
Yes	48 (17.8)	0.0584 (0.364–0.938)	0.026	0.659 (0.405–1.072)	0.093

Others genotypes were CRF85_BC,CRF55_01B,B,CRF87_cpx, URF(B/C), and URF(CRF01_AE/CRF07_BC); PDR: pretreatment drug resistance; OR: odds ratio; AOR: adjusted odds ratio; red data represent *p* ≤ 0.05.

## Data Availability

The datasets used and analyzed during this study are available from the corresponding author upon reasonable request, and with permission from Shaoxing Center for Disease Control and Prevention.

## Abbreviations

The following abbreviations are used in this manuscript:

HIV	human immunodeficiency virus
AIDS	acquired immunodeficiency syndrome
PLWHA	people living with HIV/AIDS
ART	antiretroviral therapy
WHO	World Health Organization
PDR	pretreatment drug resistance
ADR	acquired drug resistance
PCR	polymerase chain reaction
PI	Protease inhibitor
NRTI	Nucleoside reverse transcriptase inhibitor
NNRTI	Non-nucleoside reverse transcriptase inhibitor
ATV/r	Atazanavir/ritonavir
FPV/r	Fosamprenavir/ritonavir
IDV/r	Indinavir/ritonavir
LPV/r	Lopinavir/ritonavir
NFV	Nelfinavir
SQV/r	Saquinavir/ritonavir
TPV/r	Ritonavir/ritonavir
ABC	Abacavir
AZT	Zidovudine
D4T	Stavudine
DDI	Didanosine
FTC	Emtricitabine
3TC	Lamivudine
TDF	Tenofovir
DOR	Doravirine
EFV	Efavirenz
ETR	Etravirine
NVP	nevirapine
RPV	Rilpivirine
BIC	Bictegravir
TAF	Tenofovir Alafenamide
DTG	Dolutegravir

## References

[1] WHO WHO Releases HIV Drug Resistance Report 2021. https://www.who.int/news/item/24-11-2021-who-releases-hiv-drug-resistance-report-2021.

[2] Macdonald V., Mbuagbaw L., Jordan M.R., Mathers B., Jay S., Baggaley R., Verster A., Bertagnolio S. (2020). Prevalence of pretreatment HIV drug resistance in key populations: A systematic review and meta-analysis. J. Int. AIDS Soc..

[3] Buliotti S., Tadey L., Gili A., Cangelosi D., Kademian S., Cuffresi C., Salomón H., Quarleri J. (2023). Gender disparities in HIV transmitted drug resistance: Evidence of rising resistance among women of childbearing potential in Argentina. Clin. Microbiol. Infect..

[4] Chen H., Hao J., Hu J., Song C., Zhou Y., Li M., Chen J., Liu X., Wang D., Xu X. (2023). Pretreatment HIV Drug Resistance and the Molecular Transmission Network Among HIV-Positive Individuals in China in 2022: Multicenter Observational Study. JMIR Public Health Surveill..

[5] Liu X., Wang D., Hu J., Song C., Liao L., Feng Y., Li D., Xing H., Ruan Y. (2023). Changes in HIV-1 Subtypes/Sub-Subtypes, and Transmitted Drug Resistance Among ART-Naïve HIV-Infected Individuals—China, 2004–2022. China CDC Wkly..

[6] Ye J., Dong Y., Lan Y., Chen J., Zhou Y., Liu J., Yuan D., Lu X., Guo W., Yang H. (2024). Trends and Patterns of HIV Transmitted Drug Resistance in China From 2018 to 2023. J. Infect. Dis..

[7] Chinese Center for Disease Control and Prevention (2025). The national AIDS and sexually transmitted disease epidemic in December 2024. Chin. J. AIDS STD.

[8] Zhang H., Wu P., Li J., Li M. (2023). Prevalence and analysis of acquired and transmitted integrase strand transfer inhibitor-associated HIV-1 drug resistance in Chongqing, China. Virulence.

[9] Chen M., Zhu Q., Xing H., Chen H., Jin X., Dong L., Dai J., Yang M., Yang C., Jia M. (2020). The characteristics of pretreatment HIV-1 drug resistance in western Yunnan, China. Epidemiol. Infect..

[10] Liu M., He X.Q., Deng R.N., Tang S.Q., Harypursat V., Lu Y.Q., He K., Huo Q., Yang H.H., Liu Q. (2022). Pretreatment drug resistance in people living with HIV: A large retrospective cohort study in Chongqing, China. HIV Med..

[11] Xu X., Luo L., Song C., Li J., Chen H., Zhu Q., Lan G., Liang S., Shen Z., Cao Z. (2021). Survey of pretreatment HIV drug resistance and the genetic transmission networks among HIV-positive individuals in southwestern China, 2014-2020. BMC Infect. Dis..

[12] Pang X., Tang K., He Q., Huang J., Fang N., Zhou X., Zhu Q., Wu X., Shen Z., Liang S. (2021). HIV drug resistance and HIV transmission risk factors among newly diagnosed individuals in Southwest China. BMC Infect. Dis..

[13] Cao D., Xing H., Feng Y., He T., Zhang J., Ling J., Chen J., Zhao J. (2024). Molecular transmission network analysis reveals the challenge of HIV-1 in ageing patients in China: Elderly people play a crucial role in the transmission of subtypes and high pretreatment drug resistance in developed Eastern China, 2019–2023. Virol. J..

[14] Zhang J., Guo Z., Yang J., Pan X., Jiang J., Ding X., Zhang W., Xia Y., Xu Y., Huang J. (2015). Genetic diversity of HIV-1 and transmitted drug resistance among newly diagnosed individuals with HIV infection in Hangzhou. China J. Med. Virol..

[15] Gan M., Zheng S., Hao J., Ruan Y., Liao L., Shao Y., Feng Y., Xing H. (2021). The prevalence of CRF55_01B among HIV-1 strain and its connection with traffic development in China. Emerg. Microbes Infect..

[16] Zhang J., Meng J.H., Han M., Ge Z., Li L., Liu L., Li H., Liao L., Xing H., Ruan Y. (2023). Nationwide trends and molecular epidemiology of HIV-1 drug resistance in China. Emerg. Infect. Dis..

[17] World Health Organization (2024). HIV Drug Resistance: Brief Report 2024.

[18] Zhang M., Ma Y., Wang Z., Wang G., Wang Q., Li X., Lin F., Zhang C. (2024). Prevalence and transmission of pretreatment drug resistance in people living with HIV-1 in Shanghai China, 2017–2021. Virulence.

[19] Xu Y., Shi H., Dong X., Ding C., Wu S., Li X., Zhang H., Qiao M., Li X., Zhu Z. (2023). Transmitted drug resistance and transmission clusters among ART-naïve HIV-1-infected individuals from 2019 to 2021 in Nanjing, China. Front. Public Health.

[20] Zhu M., Sun Z., Zhang X., Luo W., Wu S., Ye L., Xu K., Chen J. (2024). Epidemiological dynamics and molecular characterization of HIV drug resistance in eastern China from 2020 to 2023. Front. Microbiol..

[21] Wang D., Feng Y., Hao J., Hu H., Li F., Li J., Ruan Y., Liao L., Hu J., Song C. (2024). National and Regional Molecular Epidemiology of HIV-1—China, 2004-2023. China CDC Wkly..

[22] Li X., Li Y., Liu H., Trovão N.S., Foley B.T. (2022). The emergence and transmission dynamics of HIV-1 CRF07_BC in Mainland China. Virus Evol..

[23] Cheng Z., Yan H., Li Q., Ablan S.D., Kleinpeter A., Freed E.O., Wu H., Dzakah E.E., Zhao J., Han Z. (2022). Enhanced Transmissibility and Decreased Virulence of HIV-1 CRF07_BC May Explain Its Rapid Expansion in China. Microbiol. Spectr..

[24] Han J., Zhou Y., Ma Y., Zhu G., Zhang D., Zhu B., Cheng T., Wang L., Wang J., Li L. (2022). A New HIV-1 K28E32-Reverse Transcriptase Variant Associated with the Rapid Expansion of CRF07_BC among Men Who Have Sex with Men. Microbiol. Spectr..

